# Prevalence and predictors of undiagnosed type 2 diabetes and pre-diabetes among adult Egyptians: a community-based survey

**DOI:** 10.1186/s12889-023-15819-0

**Published:** 2023-05-25

**Authors:** Hassan Farag Mohamed Farag, Ehab Elrewany, Basem Farouk Abdel-Aziz, Eman Anwar Sultan

**Affiliations:** 1 Department of Tropical Health, High Institute of Public Health, Alexandria University, Alexandria, Egypt; 2 Department of Health Administration and Behavioral Sciences, High Institute of Public Health, Alexandria University, Alexandria, Egypt; 3 Department of Community Medicine, Faculty of Medicine, Alexandria University, Alexandria, Egypt

**Keywords:** T2DM, Undiagnosed DM, PDM, Prevalence, AUSDRISK Arabic Version, Screening, Sensitivity, Specificity

## Abstract

**Background:**

The global prevalence of abnormal glycemic level comprising diabetes mellitus (DM) and pre-diabetes (PDM) is rapidly increasing with special concern for the entity silent or undiagnosed diabetes; those unaware of their condition. Identification of people at risk became much easier with the use of risk charts than the traditional methods. The current study aimed to conduct a community-based screening for T2DM to estimate the prevalence of undiagnosed DM and to assess the AUSDRISK Arabic version as a predictive tool in an Egyptian context.

**Methods:**

A cross-sectional study was conducted among 719 Adults aging 18 years or more and not known to be diabetics through a population-based household survey. Each participant was interviewed to fill demographic and medical data as well as the AUSDRISK Arabic version risk score and undergo testing for fasting plasma glucose (FPG) and oral glucose tolerance test (OGTT).

**Results:**

The prevalence of DM and PDM were 5% and 21.7% respectively. The multivariate analysis revealed that age, being physically inactive, history of previous abnormal glycemic level and waist circumference were the predictors for having abnormal glycemic level among the studied participants. At cut off points ≥ 13 and ≥ 9, the AUSDRISK respectively discriminated DM [sensitivity (86.11%), specificity (73.35%), and area under the curve (AUC): 0.887, 95% CI: 0.824–0.950] and abnormal glycemic level [sensitivity (80.73%), specificity (58.06%), and AUC: 0.767, 95% CI: 0.727–0.807], *p* < 0.001.

**Conclusions:**

Overt DM just occupies the top of an iceberg, its unseen big population have undiagnosed DM, PDM or been at risk of T2DM because of sustained exposure to the influential risk factors. The AUSDRISK Arabic version was proved to be sensitive and specific tool to be used among Egyptians as a screening tool for the detection of DM or abnormal glycemic level. A prominent association has been demonstrated between AUSDRISK Arabic version score and the diabetic status.

## Introduction

Diabetes mellitus (DM) is a metabolic disorder of multiple etiology characterized by chronic hyperglycemia with disturbances of carbohydrate, fats and protein metabolism resulting from defects in insulin secretion, insulin action or both [1].

Its global prevalence is rapidly increasing. According to the International Diabetic Federation (IDF), it was estimated that nearly 537 million adults (10.5%) all over the world suffer from DM in 2021. Almost one in two (240 million; 44.7%) adults with diabetes are unaware that they have the condition (undiagnosed diabetes) and nearly 90% of diabetic cases are type 2 DM (T2DM). This global estimate is expected to rise to 643 million (11.3%) in 2030 and to 783 million (12.2%) by 2045. The majority of diabetic patients (75%) had their residence in low and middle income countries (LMICs). [1, 2] Along with DM, the magnitude of pre-diabetes (PDM) in the form of impaired glucose tolerance (IGT) and impaired fasting glucose (IFG) is rising worldwide. According to the IDF, worldwide there were 860 million pre-diabetic adults in 2021 (16.8%) and projected to be 992 million (17.5%) and 1171 million (18.3%) by 2030 and 2045 respectively. Those persons are at a very high risk of developing T2DM. As regards Egypt, it was estimated that 10.9 million had diabetes and this number is expected to rise to 13 million by 2030 and to 20 million by 2045. This makes Egypt to rank in the 10th position among countries with highest prevalence of DM and is expected to be in the 9th position by 2045 [2–4].

Multiple modifiable (such as; obesity, sedentary lifestyle, smoking, high blood pressure and unhealthy diet consumption) and non-modifiable (like; age, ethnicity and family history) risk factors result in the development and progress of T2DM. [5] Those risk factors gave the chronic nature of the disease with a long asymptomatic period. This gave the opportunity to identify those individuals who are likely to have DM while being asymptomatic through screening program for further prophylactic intervention. It was proved by the IDF that the onset of T2DM can be delayed or even prevented through lifestyle modification by physical activity and/or healthy diet. [6]

Screening for early detection of T2DM was set to be a major area of interest by the World Health organization (WHO) and IDF since 2003. This was justified by multiple reasons such as the large proportion of people that are unaware of having DM (undiagnosed DM), the rising prevalence of DM and PDM worldwide, its long asymptomatic latency prior to its clinical overtness give an ample opportunity for its complications, the micro-vascular ones in particular, to occur in a good portion of patients before its diagnosis and the evidence-based efficacy of the intensive control of the blood glucose in patients to break the progression of DM complications. [7–9]

Screening is the backbone of T2DM preventive strategy. It aims to screen the asymptomatic apparently healthy people to find undiagnosed T2DM, PDM and those who are vulnerable (at risk) to get T2DM followed by appropriate non-pharmaceutical and/ or pharmaceutical intervention to prevent or delay its occurrence. At first screening programs for DM were exclusively one step that implies direct testing by the common invasive, inconvenient, and expensive laboratory tests including the 2-hour oral glucose tolerance test (OGTT), fasting plasma glucose (FPG), or the glycated hemoglobin (HbA1c). A two-step screening has recently evolved and get more popular worldwide. In the first step, the individuals are pre-screened using either risk scoring questionnaires to distinguish those at risk who will undergo diagnostic lab testing in the second step. [10]

Several non-invasive screening risk score charts have been developed and proved to be feasible, less time consuming, and cost effective in detecting T2DM in comparison to the traditional screening that relied for long time on invasive, inconvenient, and expensive techniques including blood sampling. [11, 12] The Finnish Diabetes Risk Score (FINDRISC) has been widely adopted in many European countries and proved to be a valid inexpensive risk assessment tool for T2DM. The German Diabetes Risk Score (GDRS) is another model for identification of individuals at high risk for T2DM and screening for undiagnosed DM, in the German population. [13, 14]

The Australian type 2 Diabetes Risk assessment tool (AUSDRISK) was developed by the Australian Commonwealth Department of Health and Ageing in 2008 to estimate the probability of a person developing T2DM within the next five years based on multiple risk factors [15]. It was rec alibrated into Arabic language and the AUSDRISK Arabic version was proved to be useful in an Egyptian context as a valid and reliable predictive tool. [16]

The bottom line is that T2DM is an ever-growing, ever-expanding disease of chronic natural history preceded by a long asymptomatic period and causes a variety of debilitating micro-vascular and macro-vascular complications that adversely affect individual health and productivity. The current study refers to the community-based screening as an invaluable approach to reach the apparently healthy individuals who are at risk of T2DM for early appropriate non-pharmaceutical and pharmaceutical intervention.

## Methods

The current study aimed to conduct a community-based screening for T2DM to estimate the prevalence of undiagnosed DM and to assess the AUSDRISK Arabic version as a screening tool among Egyptians.

A cross-sectional study was conducted at Damanhur district of El Behera Governorate in Egypt. Based on the prevalence of undiagnosed DM was about 5% [2]. A minimum required sample of 706 adults was required to detect a sensitivity with 81.3% of AUSDRISK. [17] By using precision = 5% with alpha error = 0.05. [18] 719 adults were eligible for the participation of this study aging 18 years or more and not known to be diabetics (as revealed by history taking) and recruited to participate in the study with exclusion of pregnant females or those who had advanced decompensated organ disease.

Sampling approach was adopted at first using simple random sampling, Damanhur district was selected among the 15 districts of El Behera governorate. Then WHO multistage cluster sampling technique was adopted in Damanhur district through the identification of 30 clusters to be involved in the study. Proportional allocation was used in selecting the clusters whereby two thirds (20 clusters) and one third (10 clusters) were rural and urban clusters respectively as adult population in rural areas is approximately double that of urban ones [19]. From each cluster ten households were selected and involved in the study. The selection method was started at the center of each selected cluster. The direction of selection was randomly determined using rotation bottle. The first household was randomly selected as well (as the first person came out). The second household would be the next one with an entrance adjacent to the first selected one and so on. All eligible adults within each household were included in the study. The number of adults per household ranged from 1 to 4.

Each participant was interviewed face to face to fill: (a) Demographic data entailing age, sex, marital status, education level, and residence. (b) Habitual data comprising smoking, physical activity and fruits/vegetables consumption. (c) Medical data upon abnormal lipid profile or hypertension and family history of DM. (d) The AUSDRISK Arabic version which contain 9 questions including the age, the gender, family history of DM, previous history of high blood sugar level, history of hypertension, daily smoking, vegetables and fruits intake, daily physical activity and the measurement of waist circumference. The total score of the AUSDRISK Arabic version was scaled as the following categories (mild risk: ≤ 4 points, moderate risk: 5–10 points and severe risk: ≥ 11 points). [16]

Then they were invited to perform: (a) Anthropometric measurements: weight, height, body mass index (BMI), and waist circumference. BMI is calculated as body weight in kilograms divided by the square of the height in meters (kg/m^2^) to be fallen into one of the following categories (underweight: <18.5 kg/m^2^, normal weight: 18.5–24.9 kg/m^2^, overweight (pre-obesity): 25-29.9 kg/m^2^, obesity class I: 30-34.9 kg/m^2^, obesity class II: 35-39.9 kg/m^2^ and obesity class III: ≥40 kg/m^2^). [20] (b) Fasting plasma glucose (FPG); plasma glucose level after no caloric intake for at least 8 h. (c) Oral glucose tolerance test (OGTT); plasma glucose level 2 h after intake of 75 g anhydrous glucose dissolved in water used as a glucose load. Participants were diagnosed as diabetics if FPG was ≥ 126 mg/dl or OGTT was ≥ 200 mg/dl and as pre-diabetics if FPG was 110–125 mg/dl or OGTT was 140–199 mg/dl. [21] Participants with pre-diabetes or diabetes were referred to a specialist of internal medicine for management.

### Statistical analysis

The collected data were coded, revised, cleaned, tabulated and analyzed through IBM SPSS Statistics version 26 using appropriate statistics [22]. The descriptive statistics including percentages, arithmetic mean and standard deviation (SD) were calculated for various qualitative and quantitative data to describe the study population. The analytic statistical tests comprised Chi squared and student t test. Multivariate logistic regression analysis was done for the studied variables with *p* value < 0.1 to determine the predictors for having abnormal glycemic level. The receiver operating characteristic curves (ROC) and area under the curve (AUC) were drawn to determine an optimal cutoff point of the AUSDRISK Arabic version to diagnose both DM and abnormal glycemic level and calculate its sensitivity and specificity. *P* value equal to or less than 0.05 was considered statistically significant.

## Results

The socio-demographic features showed that most of the participants were female (69.3%), with mean age of 39.36 ± 14.77 years, married (77.1%) and living in rural areas (67.3%). While 27.8% of the participants were illiterate / Write and read, 25.6% and 40.1% of them completed their higher and middle education, respectively. As regards the habits of the studied participants most of them were nonsmokers (88.3%), physically active (60.5%) and reported eating vegetables or fruits on daily basis (70%). The medical history of the studied participants revealed that 7.2% had history of abnormal glycemic level, 3.1% had abnormal lipid profile, 12.4% had hypertension and 40.3% reported positive family history of DM. [Table 1]

**Table 1:** Socio-demographic, habitual and medical characteristics of the study participants

		Total (n = 719)	
		N	%
**Age (years)**	Min-Max	18–88	
	Mean ± SD	39.36 ± 14.77	
**Gender**	Male	221	30.7
	Female	498	69.3
**Marital status**	Single	126	17.5
	Married	554	77.1
	Divorced	7	1.0
	Widowed	32	4.4
**Education level**	Illiterate / Write and read	200	27.8
	Low (primary and preparatory)	47	6.5
	Middle (secondary and technical diploma)	288	40.1
	High (university)	184	25.6
**Residence**	Urban	235	32.7
	Rural	484	67.3
**Physical activity**	Physically inactive < 2.5 h / week	284	39.5
	Physically active ≥ 2.5 h / week	435	60.5
**Smoking**	No	635	88.3
	Yes	84	11.7
**Eating fruits or vegetables**	Not on daily basis	216	30.0
	On daily basis	503	70.0
**Medical history of**:	Abnormal glycemic level	52	7.2
	Abnormal lipid profile	22	3.1
	Hypertension	89	12.4
**Family history of DM**	No	429	59.7
	Yes	290	40.3

**Fig. 1:**
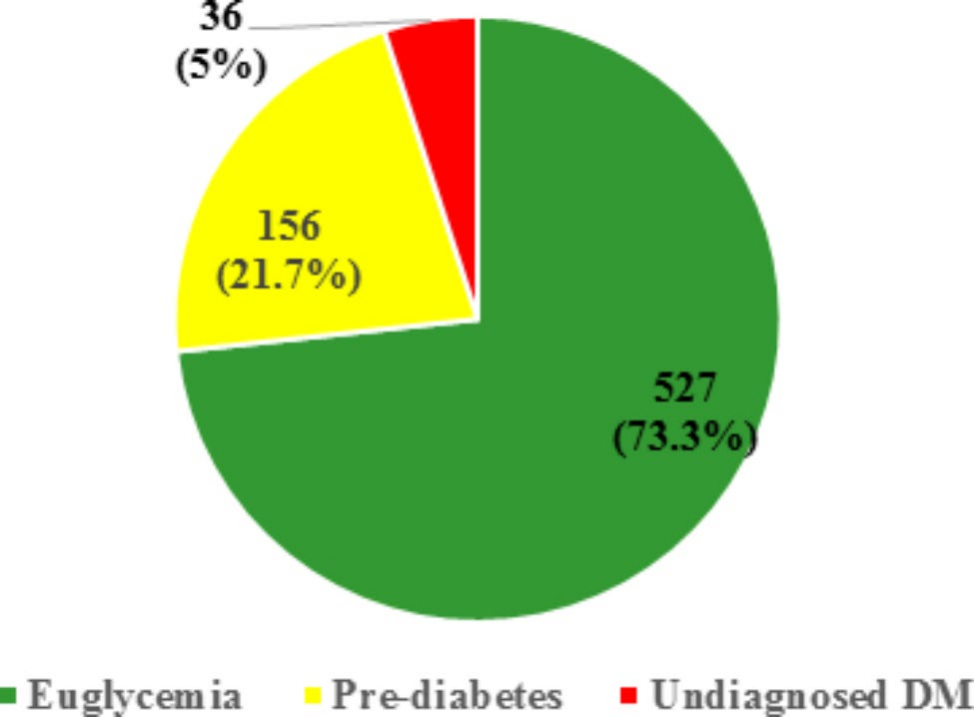
Studied participant glycemic status based on the WHO criteria

Studied participants overall anthropometric measurements recorded average of 95.42 ± 12.84 cm for waist circumference and 29.28 ± 5.52 kg/m^2^ for BMI. The BMI frequency was headed by overweight (37.4%) followed by obesity I (27.3%), and normal weight (21.4%). Most of the participants were euglycemic (73.3%, CI: 69.9–76.5%), to a lesser extent pre-diabetic (21.7%, CI: 18.7–24.9%) and the prevalence of undiagnosed DM was 5%, CI: 3.5–6.9%]. The average glycemic level was 92.02 ± 20.43 mg/dl for FPG and 121.54 ± 37.18 mg/dl for OGTT. According to the AUSDRISK Arabic version, 39.4% and 39.1% had moderate and high risk for developing DM while only 21.5 had mild risk. [Table 2; Fig. 1]

**Table 2:** Studied participant anthropometric measurements, levels of glycemia and AUSDRISK Arabic version score

		Total (n = 719)	
		Min – Max	Mean ± SD
**Anthropometric measures**	Waist circumference (cm)	60–133	95.42 ± 12.84
	Body mass index (kg/m^2^)	18.55–57.87	29.28 ± 5.52
**Plasma glucose level**	Fasting	69–302	92.02 ± 20.43
	2-hour plasma glucose level (OGTT)	81–443	121.54 ± 37.18
**Body mass index categories**		**N**	**% (CI)**
Normal weight		154	21.4 (18.5–24.6)
Overweight		269	37.4 (33.9–41.1)
Obese I		196	27.3 (24.0-30.7)
Obese II		71	9.9 (7.8–12.3)
Obese III		29	4.0 (2.7–5.7)
**AUSDRISK Arabic version score**	Mild risk ≤ 4	155	21.5 (18.6–24.7)
	Moderate risk 5–10	283	39.4 (35.8–43.0)
	High risk ≥ 11	281	39.1 (35.5–42.8)

Age owed significant relationship with having abnormal glycemic level. There was significant increment in the mean age among participants having abnormal glycemic level (46.85 ± 14.95) compared to euglycemia (36.64 ± 13.73) (*p* < 0.001). Salient association of having abnormal glycemic level to lower education among illiterate / read and write (35.5%) and low education level (21.3%) in comparison to the middle (19.4%) ones, *p* = 0.001. Also urban residency showed higher significant frequency for having abnormal glycemic level (31.5%) than rural areas (24.4%), *p* = 0.043. Higher frequency having abnormal glycemic level among physically inactive participants (38%) than physically active ones (19.3%) was evident, the differences in between reached significant level, *p* < 0.001. The recall revealed significant linkage of having abnormal glycemic level to the previous history of high blood sugar (88.8% vs. 21.9%), positive history of hypertension (50.6% vs. 23.3%), and positive family history of DM (34.1% vs. 21.7%) with *p* < 0.001. The average waist circumference was significantly higher among those having abnormal glycemic level (101.4 ± 12.92 cm) than the euglycemic state (93.25 ± 12.11 cm), *p* < 0.001. Significant association to BMI was observed where the prevalence of abnormal glycemic level was higher among obese (39.5%) and overweight (20.1%) in comparison to normal weight (13.6%), *p* < 0.001. None of the gender, smoking, the intake of fruits and vegetables, recall history of abnormal lipid profile had statistically significant connection to the diabetes status. [Table 3]

**Table 3:** Relationship between having abnormal glycemic level and the studied variables among the participants

		Glycemic level N (%)		Statistical Test (P-value)
		Normal (n = 527)	Abnormal (n = 192)	
**Age (years)**	Min-Max	18–88	18–85	t = -8.614^*^ *p* < 0.001
	Mean ± SD	36.64 ± 13.73	46.85 ± 14.95	
**Gender**	Male (n = 221)	169 (76.5)	52 (23.5)	χ^2^ = 1.643 *p* = 0.2
	Female (n = 498)	358 (71.9)	140 (28.1)	
**Education level**	Illiterate / Write and read (n = 200)	129 (64.5)	71 (35.5)	χ^2^ = 17.323^*^ *p* = 0.001
	Low (primary and preparatory) (n = 47)	37 (78.7)	10 (21.3)	
	Middle (secondary and technical diploma) (n = 288)	232 (80.6)	56 (19.4)	
	High (university) (n = 184)	129 (70.1)	55 (29.9)	
**Residence**	Urban (n = 235)	161 (68.5)	74 (31.5)	χ^2^ = 4.085^*^ *p* = 0.043
	Rural (n = 484)	366 (75.6)	118 (24.4)	
**Smoking**	No (n = 635)	460 (72.4)	175 (27.6)	χ^2^ = 2.031 *p* = 0.154
	Yes (n = 84)	67 (79.8)	17 (20.2)	
**Physical activity**	Physically inactive < 2.5 h / week (n = 284)	176 (62.0)	108 (38.0)	χ^2^ = 30.756^*^ *p* < 0.001
	Physically active ≥ 2.5 h / week (n = 435)	351 (80.7)	84 (19.3)	
**Eating fruits or vegetables**	Not on daily basis (n = 216)	149 (69.0)	67 (31.0)	χ^2^ = 2.937 *p* = 0.087
	On daily basis (n = 503)	378 (75.1)	125 (24.9)	
**History of abnormal glycemic level**	No (n = 667)	521 (78.1)	146 (21.9)	χ^2^ = 109.228^*^ *p* < 0.001
	Yes (n = 52)	6 (11.5)	46 (88.5)	
**History of abnormal lipid profile**	No (n = 697)	514 (73.7)	183 (26.3)	χ^2^ = 2.34 *p* = 0.126
	Yes (n = 22)	13 (59.1)	9 (40.9)	
**History of hypertension**	No (n = 630)	483 (76.7)	147 (23.3)	χ^2^ = 29.539^*^ *p* < 0.001
	Yes (n = 89)	44 (49.4)	45 (50.6)	
**Family history of diabetes**	No (n = 429)	336 (78.3)	93 (21.7)	χ^2^ = 13.724^*^ *p* < 0.001
	Yes (n = 290)	191 (65.9)	99 (34.1)	
**Waist circumference (cm)**	Min-Max	60–133	65–132	t = -7.836^*^ *p* < 0.001
	Mean ± SD	93.25 ± 12.11	101.4 ± 12.92	
**BMI**	Normal weight (n = 154)	133 (86.4)	21 (13.6)	χ^2^ = 44.343^*^ *p* < 0.001
	Over weight (n = 269)	215 (79.9)	54 (20.1)	
	Obese (n = 296)	179 (60.5)	117 (39.5)	

t: Calculated value for Student t test

χ^2^: Calculated value for Chi square test

*: Statistically significant at *p* ≤ 0.05

Multivariate analysis revealed that age, being physically inactive, history of previous abnormal glycemic level and waist circumference were the predictors for having abnormal glycemic level among the studied participants [Table 4].

**Table 4:** Multivariate logistic regression analysis for predictors of having abnormal glycemic level among the participants

		Adjusted OR	95% CI; LL-UL	*p* value
**Age (years)**		1.028	1.012–1.045	< 0.001^*^
**Education level**	Illiterate / Write and read	0.834	0.431–1.616	0.591
	Low	0.591	0.227–1.54	0.281
	Middle	0.93	0.531–1.629	0.799
**Urban residence**		1.283	0.739–2.228	0.376
**Sedentary lifestyle (< 2.5 h / week)**		1.653	1.056–2.586	0.028^*^
**Not eating fruits or vegetables on daily basis**		1.175	0.752–1.834	0.479
**History of abnormal glycemic level**		24.361	9.378–63.28	< 0.001^*^
**History of hypertension**		1.308	0.735–2.330	0.362
**Family history of diabetes**		1.361	0.916–2.024	0.127
**Waist circumference (cm)**		1.024	1.004–1.046	0.021^*^
**BMI**	Over weight	1.097	0.579–2.077	0.776
	Obese	1.497	0.736–3.045	0.265

OR: Odds ratio

CI: Confidence interval

LL: Lower limit

UL: Upper limit

*: Statistically significant at p ≤ 0.05

**Fig. 2:**
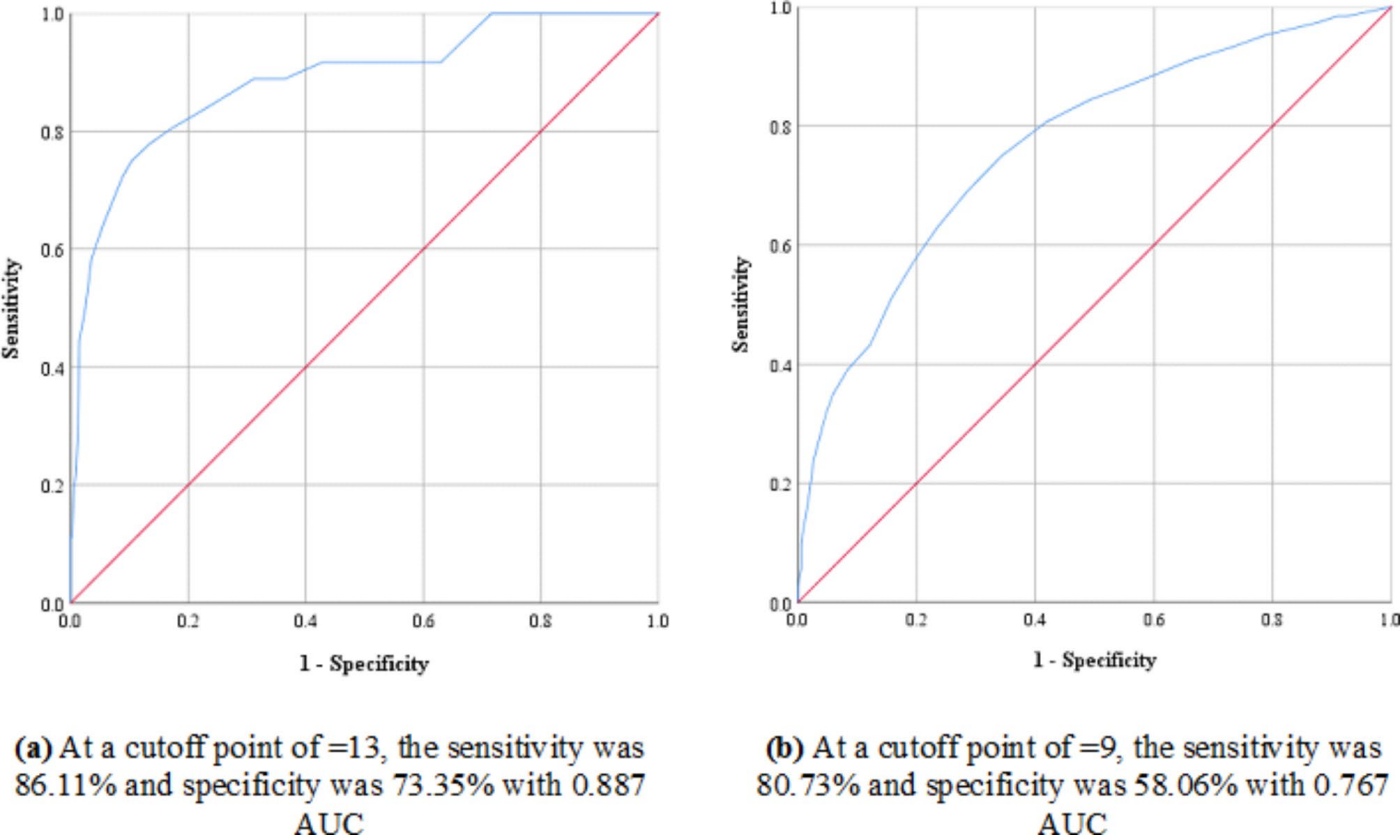
ROC curve for the AUSDRISK Arabic version as a screening diagnostic tool for DM (**a**) and abnormal glycemic level (**b**) AUC: Area under the curve ROC: Receiver operating characteristic curve DM: Diabetes mellitus

At a cutoff point of ≥ 13, the sensitivity and specificity of the AUSDRISK Arabic version for detection of undiagnosed DM were 86.11% and 73.35 respectively with 0.887 (confidence interval (CI): 0.824–0.95) area under the curve (AUC), *p* < 0.001 While the sensitivity and specificity for detection of abnormal glycemic level were 80.73% and 58.06% respectively at a cutoff point ≥ 9 with 0.767 AUC (CI: 0.727–0.807), *p* < 0.001. [Figure 2]

A dual statistical confirmation of a prominent association between diabetic state and AUSDRISK Arabic version score was proved. DM and PDM participants had a significant higher averaged total AUSDRISK score (19.78 ± 6.44, 12.59 ± 5.53) than the euglycemic ones (7.98 ± 5.09) with F value of 117.419 (P < 0.01). The percentage of DM and PDM increased significantly with AUSDRISK score ≥ 11 (11.4%, 35.6%) compared to the AUSDRISK score 5–10 (1.4%, 15.2%) and AUSDRISK score ≤ 4 (0.00%, 8.4%); X^2^ = 107.854 (P < 0.01). [Table 5]

**Table 5:** Relationship between studied participant AUSDRISK Arabic version scores and their glycemic status

	Glycemic status						Statistical Test (P-value)
	Euglycemia (n = 527)		PDM (n = 156)		DM (n = 36)		
**AUSDRISK Arabic version score**	**N**	**%**	**N**	**%**	**N**	**%**	χ^2^ = 107.854* (*p* < 0.01)
Mild risk ≤ 4	142	91.6	13	8.4	0	0.00	
Moderate risk 5–10	236	83.4	43	15.2	4	1.4	
High risk ≥ 11	149	53.0	100	35.6	32	11.4	
Min-Max	0–26		0–27		6–31		ANOVA = 117.419* (*p* < 0.01)
Mean ± SD	7.98 ± 5.09		12.59 ± 5.53		19.78 ± 6.44		

χ^2^: Calculated value for Chi square test

*: Statistically significant at *p* ≤ 0.05

## Discussion

The burden of T2DM imputes, in part, to its high prevalence. Commonness of T2DM latency and silence adds further burden as they cumulate opportunistic risk for evolution of its micro-vascular and macro-vascular complications. Latency refers to the transition period of PDM that lapses prior to its conversion to T2DM which often takes several years. Silent T2DM represents the undiagnosed/ undetected T2DM. The frequency of undiagnosed T2DM has been noted to reach as high as 50% of patients; they got diagnosed incidentally. In 2019, in the list of top 10 countries with highest number of undiagnosed DM, Egypt booked the eighth seat by 4.8 million adults (20–79 years) not aware of being diseased. [4]

The variability in the prevalence of DM and PDM seen worldwide is multifactorial. Among the implicated factors are the variation in the study type, approach, site, sample size, sampling technique, the diagnostic criteria of glycemic status, characteristics of the involved population (age, gender, ethnicity, etc.). Of note is that high prevalence of undiagnosed DM which reach almost half of the diabetic cases around 5% was a common noteworthy trait in the vast majorities of the studies [2]. In the current study, the biochemical results showed that 73.3%, 21.7% and 5% of the participants had blood glucose levels indicative of euglycemia, PDM, and DM, respectively. It was well evident that 5% of the participants were newly undiagnosed T2DM as none of them was aware of the disease. Close figures for the prevalence of undiagnosed DM have been reported worldwide. In Alexandria, Egypt, a recent cross-sectional study reported a 5.5% age-adjusted prevalence of undiagnosed DM in a sample of 9657 adults aged 18–90 years while delineating the epidemiological profile of DM. [23] A similar prevalence of undiagnosed DM of 5.6% was identified in another cross-sectional study carried out to profile the metabolic syndrome in a sample of 270 adults (> 20 years) recruited from rural and urban districts of Alexandria. [24] Akin undetected DM frequencies of 5.7% and 4.9% were respectively inferred in two separate community-based cross-sectional studies done to investigate the prevalence and risk factors of DM among 402 adults aged 15 + years in Mizan-Aman town, southwest Ethiopia and 587 adults (18 + years) in Dessie Town, Northeast Ethiopia. [25, 26] In Asia, similar findings were reported in a national survey of 18,066 adult Thai population to determine the prevalence of DM; 4.1% of the participants revealed glycemic status consistent with DM. [27]

The increasing prevalence of PDM worldwide is alarming. It constitutes a highly significant expanding reservoir of the futuristic T2DM albeit good proportion of the PDM restore their euglycemia and don’t progress to overt DM. Known DM represents the tip of the iceberg of impaired glucose metabolism where the vast majority of PDM lurks unseen. High prevalence of PDM (latent T2DM) was a worthy finding in the current study where 21.7% of the recruited participants had PDM and were still in the transition period. Comparable with the result of the current study were the elevated prevalence of PDM of 22.4% and 22.3% respectively seen in Peru national population-based survey and the result of a recent meta-analysis. [28, 29] Nevertheless, lower prevalence of PDM of around 15% have been recorded in multiple studies in Egypt and Ethiopia. [23–26]

The uprising incidence, and prevalence of DM and PDM worldwide didn’t originate from space. It has been strongly attributed to expansion of the avoidable risk factors and the urbanized lifestyle. The American Diabetes Association (ADA) and the National Institute of Diabetes and Digestive and Kidney Diseases have enlisted a set of 11 T2DM and PDM major risk factors. It included 3 unmodifiable; namely are the age 45 + years; risky races/ ethnicities and family history of DM as well as 8 modifiable ones. Those latter are the overweight/ obesity; hypertension or on anti-hypertensive medications; physical inactivity; history of gestational diabetes (GDM) or giving birth of an overweight baby (weight: 9 + pounds), dyslipidemia of low HDL-cholesterol or hypertriglyceridemia; history of cardiovascular stroke; polycystic ovary syndrome and acanthosis nigricans. [30, 31]

In the current study, the univariate analysis revealed 9 risk factors comprising the age, level of education, urban residence, physical inactivity, medical history of high glucose level, history of hypertension, family history of DM as well as the overweight/ obesity and waist circumference, while the multivariate analysis revealed that age, being physically inactive, history of previous abnormal glycemic level and waist circumference were the predictors for having abnormal glycemic level among the studied participants. In agreement with the present results, in Egypt recently it was confirmed an increase in the prevalence of DM among the old age group. In the same vein, the national survey in Thailand, affirmed higher prevalence of DM, undiagnosed DM and PDM in the old age groups in both gender. [23, 27] An inextricable cohesion between DM and ignorance has been frequently proved everywhere. This was seen in Peru where it was asserted a statistically significant association between DM and lower education level in comparison to the higher educational ones. [28] Further proof was illustrated also in Thailand and Egypt. [23, 27] Likewise, the beneficial impact of physical exercise on the glycemic status was robustly shown in recent reviews. The significant benefit of daily 30-minute walking was spotlighted in the control of glycemia, lowering the risk of T2DM by ≈ 50%, decreasing the risk of cardiovascular stroke and its consequent mortality and being a key ingredient of the treatment plan of T2DM. [31–33] It is obvious that physical activity did not monopolize the protection against DM development; it used to share it with vegetables and fruits intake abreast. [34, 35] Again in Egypt and Ethiopia, it was endorsed an eminent closeness of DM frequency towards the positive side of the history of hypertension and family history of DM rather than the negative one. [23, 25] In accordance with other studies, the present study provided a titanic testimony to the rationale of the verdict accusing history of dysglycemia as the prime suspect in the causation of having abnormal glycemic level. [36–38] Higher frequency of T2DM in overweight/ obese participants than the normal weight ones has been evidenced in different cross-sectional studies. [23–26, 39] Diabesity is the term adopted to describe the strong co-incidence between T2DM and obesity. [40]

The AUSDRISK Arabic version was proved to be sensitive and specific tool to be used among Egyptians as a screening tool. [16] It was accurate enough in the current study to discriminate undiagnosed T2DM at a cutoff point ≥ 13, (AUC: 887, sensitivity: 86.11% & specificity: 73.35%) and abnormal glycemic level at a cut off > 9 (AUC: 0.767, sensitivity: 80.73% & specificity: 58.06%). According to the AUSDRISK Arabic version score, 39.4% and 39.1% had moderate and high risk for developing DM while only 21.5 had mild risk. These findings are not identical to previous studies conducted outside [15, 17, 41] and inside [24] Egypt in which 50% of the participants were at high T2DM risk. Involvement of younger age group stood behind this discrepancy as the older the age the more the AUSDRISK score and higher the risk of T2DM and vice versa. Again, the young age caused the lower proportion of participants at high T2DM risk among Australians in a study to predict the risk of T2DM, [42] and among the Egyptian and Malaysian students at Tanta University, in Egypt which brought to light the simplicity and practicality of the AUSDRISK screening power [43].

Derivation, Modeling, and internal validation of the AUSDRISK was launched in 2010 in the Australian diabetes obesity and lifestyle intervention (AusDiab) long-term project through a nested biphasic study. Risk factors have been converted into a feasible, non-invasive, and accurate predictive AUSDRISK model with a cut-off point at 12, AUC, sensitivity and specificity, 0.78, 74% and 67.7% respectively. [15] Reliability of the AUSDRISK screening potentiality for characterizing people at high T2DM risk for further lifestyle change has been tested and verified by a cross-sectional study. At cutoff value of the AUSDRISK ≥ 12, the sensitivity and specificity were 81.3% and 57.7 respectively. [17] Employment of the AUSDRISK to early detect T2DM in a sample of 40–59 year villagers was carried out in a cross-sectional study in Indonesia. It characterized successfully the participants with scores below and above the AUSDRISK cut off level (12 points) with as high as sensitivity and specificity of 93.46% and 70.98% respectively. [44]

The study has a limitation of being restricted to only one Egyptian Governorate, namely, El Behera Governorate. To be generalizable to the overall Egyptian situation, the study needs to be replicated to represent the 27 Egyptian Governorates. Generalizability to the Arab World needs extending the study to represent the 22 member countries of the Arab league. Generalizability to Arab speaking communities all over the globe needs more extensive research efforts.

## Conclusion and recommendation

Overt DM just occupies the top of an iceberg, its unseen big population have undiagnosed DM; which is not infrequent (5%), PDM; which is the big reservoir from which overt DM emerge (21.7%) or been at risk of T2DM because of sustained exposure to the influential risk factors. The main risk factors that strongly influence abnormal glycemic level were age, being physically inactive, previous history of abnormal glycemic level and waist circumference. The AUSDRISK Arabic version was proved to be sensitive and specific tool to be used among Egyptians as a screening tool for the detection of DM or abnormal glycemic level. A prominent association has been demonstrated between AUSDRISK Arabic version score and the diabetic status. It is recommended to establish a nationwide program for early detection of undiagnosed DM and those at risk of developing DM with comprehensive intervention measures for lifestyle modification. Also to apply the AUSDRISK Arabic version during opportunistic screening or mass public surveys to identify population at risk of T2DM.

## Data Availability

Data is available from the corresponding author on reasonable request.

## List of abbreviations

ADA: American Diabetes Association
AUC: Area under the curve
AUSDRISK: Australian Type 2 Diabetes Risk Assessment Tool
BMI: Body mass index
CI: Confidence interval
DM: Diabetes Mellitus
FPG: Fasting Plasma Glucose
GDM: Gestational diabetes
HbA1c: Glycated Hemoglobin
IDF: International Diabetic Federation
IGT: Impaired glucose tolerance
IFG: Impaired fasting glucose
LL: Lower limit
LMIC: Low and Middle Income Countries
OGTT: Oral Glucose Tolerance Test
OR: Odds ratio
PDM: Pre-diabetes
ROC: Receiver operating characteristic curves
SD: Standard Deviation
T2DM: Type 2 Diabetes Mellitus
UL: Upper limit
WHO: World Health Organization
